# Draft Genome Sequence and Annotation of Sporanaerobacter acetigenes Strain F-12, Isolated from a Cattle Rumen

**DOI:** 10.1128/MRA.00634-19

**Published:** 2019-07-25

**Authors:** Seungha Kang, Stuart Denman, Reza Tahmasbi, Chris McSweeney

**Affiliations:** a CSIRO Agriculture and Food, Queensland Bioscience Precinct, St. Lucia, QLD, Australia; b Department of Animal Science, Faculty of Agriculture, Shahid Bahonar University of Kerman, Kerman, Iran; University of Arizona

## Abstract

We report the genome sequence of Sporanaerobacter acetigenes strain F-12, isolated from the rumen of a steer grazing on Rhodes grass in Townsville (Lansdown Research Station), Queensland, Australia. This draft genome consists of 2,866,191 bp, with 31.23% G+C content and 2,889 predicted coding sequences.

## ANNOUNCEMENT

The type strain Sporanaerobacter acetigenes Lup 33 (= DSM 13106^T^ = CIP 106730^T^, GenBank accession no. FQXR00000000) is an acetogenic and sulfur-reducing bacterium that was isolated from an anaerobic sludge blanket reactor in Mexico (1). This strain was identified in an ovine rumen consortium which was able to detoxify soil contaminated with residues from explosives such as hexahydro-1,3,5-trinitro-1,3,5-triazine (RDX) (2). Recently, two Sporanaerobacter strains, NJN-17 and PP17-6a, were isolated from biogas sludge, and their genome sequences were released in GenBank under accession no. CP035282 (BioSample no. SAMN10743633) and FMIF00000000, respectively.

Here, we report on S. acetigenes strain F-12 and its genome analysis. This strain was isolated from the rumen of a steer grazing in Australia (Lansdown Research Station, Townsville, Queensland) using an anaerobic basal medium (3) containing 0.8% yeast extract (BD Biosciences, USA) at 39°C. Pure cultures were streaked onto solid agar medium for isolation three times until single phenotypes were obtained. All cultures were inoculated and incubated (39°C) in a Coy anaerobic chamber (CoyLab, MI, USA) inflated with an atmosphere of CO_2_ (95%) and H_2_ (5%). The genomic DNA of strain F-12 was purified using the DNeasy blood and tissue kit (Qiagen, USA), according to the protocol.

DNA sequencing was performed at Macrogen (Seoul, South Korea) on an Illumina HiSeq 2500 (2 × 100-bp paired-end) platform (South Korea) using the TruSeq Nano DNA library kit to produce 65,200,542 total reads and 22.6× coverage. Raw reads were quality trimmed and checked for adaptor contamination with Trimmomatic version 0.32 (4), followed by *de novo* assembly using the SPAdes version 3.6.0 algorithm with default parameters (5). We obtained 50 contigs (the largest one being 275,028 bp) with a total length of 2,864,795 bp (using only contigs larger than 500 bp), a G+C content of 31.23%, and an *N*_50_ value of 156,536 bp, as defined by the Quality Assessment Tool for Genome Assemblies (QUAST version 4.0 [6]), computed with the default setting. Genome annotation was done using the Rapid Annotations using Subsystems Technology (RAST) server. According to the RAST analysis, 2,896 protein-coding genes and 69 RNAs were predicted. Nine hundred ninety-five distributions of subsystem categories in RAST were assigned, and the maximum gene counts were functionally associated with the metabolism of proteins (206 coding genes), followed by amino acids and their derivatives (133 coding genes) and carbohydrates (112 coding genes).

The 16S rRNA gene sequence of strain F-12 was 99% identical to that of the type strain, *S. acetigenes* strain Lup 33, using BLAST 2 sequences (https://blast.ncbi.nlm.nih.gov/Blast.cgi). Genomes were analyzed using JSpeciesWS to calculate the average nucleotide identity values using BLAST+ (ANIb) (7), and the Genome-to-Genome Distance Calculator (GGDC) version 2.1 (8) was used to estimate *in silico* DNA-DNA hybridization (*is*DDH) values between strains. An ANIb result of 99.22% and GGDC of 96.4% *is*DDH were obtained between strains F-12 and Lup 33^T^, supporting their affiliation in the same species. However, the ANIb values of the strain F-12 with the other two strains, NJN-17 and PP17-6a, were 70.43% and 69.86%, respectively. The type strain Lup 33 is an acetogen which produces the only acetate from glucose metabolism (1). Acetate kinase (*ackA*) and phosphotransacetylase (*pta*) genes play a crucial role in the production of acetate, and the strain F-12 genome contained one copy of *ackA* and two copies of *pta* genes. Strain Lup 33^T^ facultatively uses elemental sulfur as a terminal electron acceptor, producing sulfide (1). The sulfide reductase gene (*dsrE* family), which encodes an enzyme to participate in sulfur metabolism, was annotated in the genome of strain F-12. The role of Sporanaerobacter acetigenes strain F-12 is unknown, but its role in N metabolism and the capacity of biotransformation of environmental contamination, such as that caused by RDX, in the rumen are worthy of further investigation.

### Data availability.

The draft genome sequence for Sporanaerobacter acetigenes strain F-12 has been deposited in DDBJ/EMBL/GenBank under the accession no. SETF00000000, BioProject no. PRJNA507312, and BioSample no. SAMN10856089. The raw genomic sequencing reads are available in the Sequence Read Archive (SRA) database under accession no. SRR8529233.
